# RASAL2, a RAS GTPase-activating protein, inhibits stemness and epithelial–mesenchymal transition via MAPK/SOX2 pathway in bladder cancer

**DOI:** 10.1038/cddis.2017.9

**Published:** 2017-02-09

**Authors:** Ke Hui, Yang Gao, Jun Huang, Shan Xu, Bin Wang, Jin Zeng, Jinhai Fan, Xinyang Wang, Yangyang Yue, Shiqi Wu, Jer-Tsong Hsieh, Dalin He, Kaijie Wu

**Affiliations:** 1 Department of Urology, The First Affiliated Hospital of Xi'an Jiaotong University, Xi'an 710061, China; 2 Department of Urology, Shaanxi Provincial People's Hospital, Xi'an 710068, China; 3 Department of Urology, University of Texas Southwestern Medical Center, Dallas, TX 75390, USA

## Abstract

Muscle-invasive or metastatic bladder cancer (BCa) is associated with a very poor prognosis, and the underlying mechanism remains poorly understood. In this study, we demonstrate RASAL2, a RAS GTPase-activating protein (RAS GAP), acts as a tumor suppressor in BCa. First, RASAL2 was downregulated in BCa specimens and inversely correlated with pathological grades and clinical stages. Furthermore, we observed that RASAL2 could inhibit BCa stemness and epithelial–mesenchymal transition (EMT) based on our gain-of-function and loss-of-function experiments. Mechanistically, we found that mitogen-activated protein kinase/SOX2 signaling had a critical role for maintaining the stemness and mesenchymal properties of RASAL2-deficient BCa cells because inhibition of ERK activity or knockdown of SOX2 could reverse these phenotypes. Also, RASAL2 could inhibit BCa tumorigenesis and distant metastasis *in vivo*. Moreover, there was an inverse correlation between RASAL2 expression and the stemness/EMT status in subcutaneous xenograft and human BCa specimens. Taken together, our data indicate that RASAL2 is a tumor suppressor in BCa, and modulates cancer stemness and EMT for BCa recurrence and metastasis.

Bladder cancer (BCa) is one of the most common urological cancers worldwide, and most of BCa are transitional cell carcinoma.^1^ More than 70% cases are non-muscle-invasive BCa (NMIBC), which can be treated with transurethral resection. However, >50% cases will relapse and 10–30% cases will progress into muscle-invasive BCa (MIBC), and finally result in distant metastasis and poor prognosis of patients.^2^ So it is urgent to understand the molecular mechanisms of BCa recurrence and metastasis, and explore novel therapeutic targets to improve patient survival.

Nowadays, cancer stemness has been identified in many cancer types. Cancer stem cells (CSCs) share some properties with somatic stem cells, including self-renewal and multi-potent differentiation potential.^3, 4^ Previous studies have revealed that cancer stemness is responsible for tumorigenicity, therapeutic resistance, relapse and metastasis in BCa.^5, 6^ Also, accumulating evidences suggest that epithelial–mesenchymal transition (EMT) has an important role in the enrichment of cells with CSC properties, which are believed to be the origin of cancer progression.^7, 8^ Importantly, recent whole-genome studies have discovered the intrinsic basal and luminal MIBC subtypes associated with different chemotherapy sensitivity or patient prognosis, in which a comprehensive molecular alteration involving CSC and EMT has been well characterized.^9, 10, 11^ All these findings provide new insights about invasion and metastasis of BCa.

RASAL2 is a member of RAS GTPase-activating proteins (RAS-GAPs), which can catalyze GTP into GDP and inactivate RAS.^12, 13^ Several preliminary studies have reported the aberrant expression of RASAL2 in different cancer types, including breast cancer, ovarian cancer, lung cancer and nasopharyngeal carcinoma.^14, 15, 16, 17, 18^ However, its tumor-suppressive or oncogenic roles in cancer development remain controversial,^16, 18^ especially, its expression and function in BCa are completely unknown. In this study, we will determine the dysregulation of RASAL2 expression in BCa specimens, and explore its unique roles in modulating stemness and EMT phenotypes of BCa.

## Results

### RASAL2 is downregulated in human BCa tissues and inversely correlated with tumor grades and clinical stages

**Figure 1:**
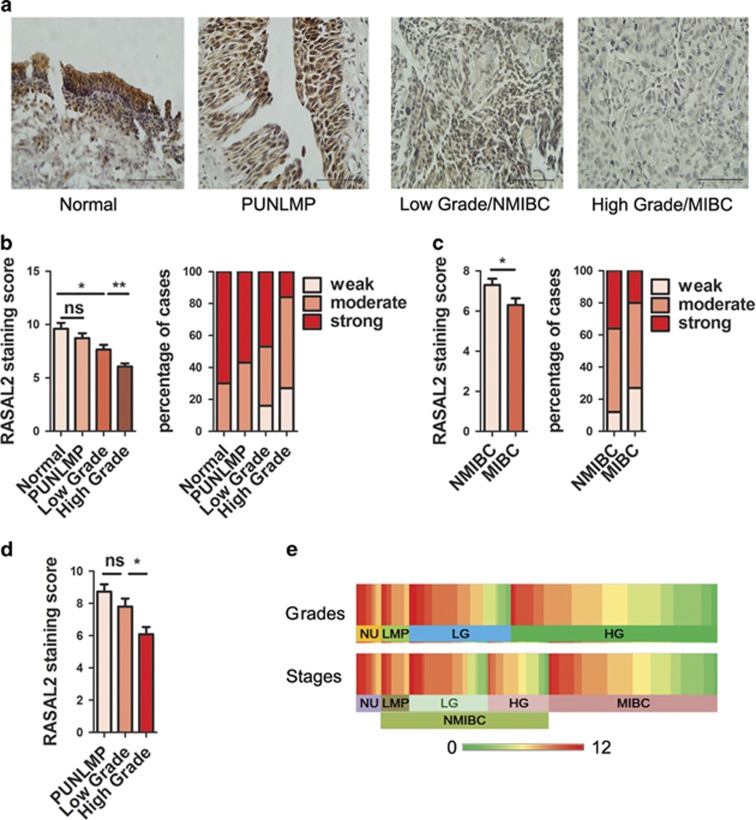
Expression of RASAL2 in BCa tissues. (**a**) Representative pictures of IHC staining of RASAL2 in normal urothelium, papillary urothelial neoplasm of low malignant potential (PUNLMP), low grade/NMIBC and high grade/MIBC. The scale bar represents 100 *μ*m. (**b** and **c**) Quantification and percentage analysis of RASAL2 staining in normal urothelium and BCa tissues with different grades (**b**) and stages (**c**) were shown. Normal urothelium (NU, *n*=10), PUNLMP (LMP, *n*=11), low-grade BCa (LG, *n*=40), high-grade BCa (HG, *n*=81), NMIBC (*n*=66) and MIBC (*n*=66). (**d**) Quantification analysis of RASAL2 staining in NMIBC tissues with different grades. (**e**) Heatmap for IHC of RASAL2 staining in BCa tissues with different grades and stages. *P<0.05, **P<0.01

To investigate the expression pattern of RASAL2 in BCa tissues, we performed immunohistochemical staining in our cohort including 132 cases of BCa and 10 cases of normal bladder epithelium specimens (Table 1). As shown in Figures 1a, b and e, we found that the expression of RASAL2 protein was not only lower in BCa tissues than normal epithelia, but also negatively correlated with tumor grades (*P<0.05, **P<0.01). Moreover, the expression of RASAL2 in MIBC tissues was significantly lower than that in NMIBC tissues (Figures 1a, c and e, *P<0.05). Furthermore, we found that RASAL2 expression in high-grade NMIBC tissues are lower than that in low-grade NMIBC tissues (Figures 1d and e, *P<0.05). Also, we analyzed RASAL2 expression in another cohort of BCa tissues profiled by microarray from NCBI GEO. Consistently, we further observed that RASAL2 mRNA was downregulated in human BCa tissues, and there was a negative correlation between RASAL2 and BCa grades and stages (Supplementary Figure 1A, *P<0.05, ***P<0.001), indicating that RASAL2 loss may be associated with BCa progression.

### RASAL2 modulates the stemness properties of BCa cells

**Figure 2:**
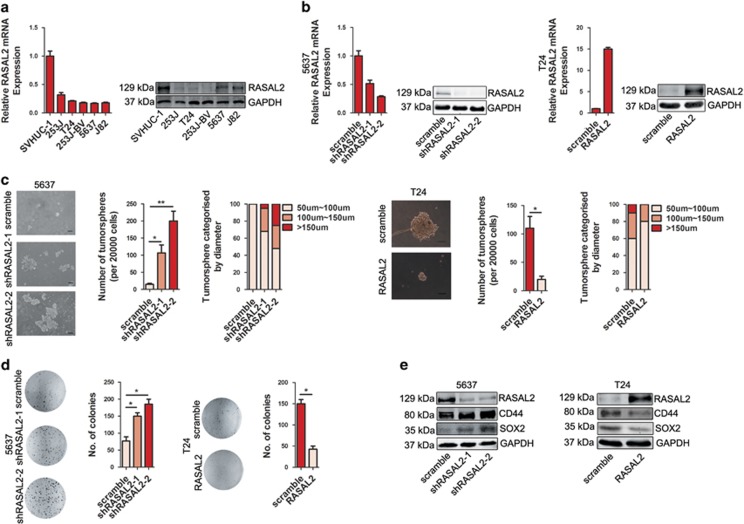
RASAL2 suppresses stemness in BCa cells. (**a**) Quantitative real-time RT-PCR and western blotting analysis of RASAL2 levels in human BCa cell lines. (**b**) Quantitative real-time RT-PCR and western blotting analysis of RASAL2 levels in 5637 cells transfected with RASAL2 shRNAs and scramble shRNA, or T24 cells infected with RASAL2 lentivirus and scramble control. (**c**) Tumorsphere assay of 5637 cells transfected with RASAL2 shRNAs and scramble shRNA, or T24 cells infected with RASAL2 lentivirus and scramble control. The tumorsphere number was counted and plotted, and percentage of tumorspheres with diameters <50 *μ*m, 50–100 *μ*m or >100 *μ*m was calculated and plotted. The scale bar represents 100 *μ*m. (**d**) Colony formation assay of 5637 cells transfected with RASAL2 shRNAs and scramble shRNA, or T24 cells infected with RASAL2 lentivirus and scramble control. The colonies number was counted and plotted. (**e**) Western blotting analysis of RASAL2, CD44 and SOX2 in 5637 cells transfected with RASAL2 shRNAs and scramble shRNA, or T24 cells infected with RASAL2 lentivirus and scramble control. GAPDH was used as internal loading control. *P<0.05, **P<0.01

Cancer stemness is one of the hotspot mechanisms leading to BCa development.^5^ Therefore, we applied both gain-of-function and loss-of-function strategies to further explore the roles of RASAL2 in BCa stemness. As screening in Figure 2a by quantitative RT-PCR and western blotting assays, RASAL2 is highly expressed in SVHUC-1, an immortalized normal bladder epithelial cell line, whereas a relatively lower expression was detected in all BCa cell lines. Fortunately, we successfully established the stable 5637 sublines with endogenous RASAL2 knockdown and T24 sublines with ectopic RASAL2 overexpression (Figure 2b). Indeed, we noticed that knockdown of RASAL2 expression in 5637 cell lines enhanced the ability of tumorsphere and colony formation, meanwhile overexpression of RASAL2 in T24 cells could decrease this ability (Figures 2c and d, *P<0.05, **P<0.01). Consistently, western blotting data clearly showed that levels of potential stemness markers SOX2 and CD44 protein increased in 5637 cells after RASAL2 knockdown, however, ectopic RASAL2 expression could downregulate SOX2 and CD44 expression in T24 cells (Figure 2e). These data indicate that RASAL2 loss could potentiate CSC phenotype of BCa cells.

### RASAL2 modulates EMT in BCa cells

**Figure 3:**
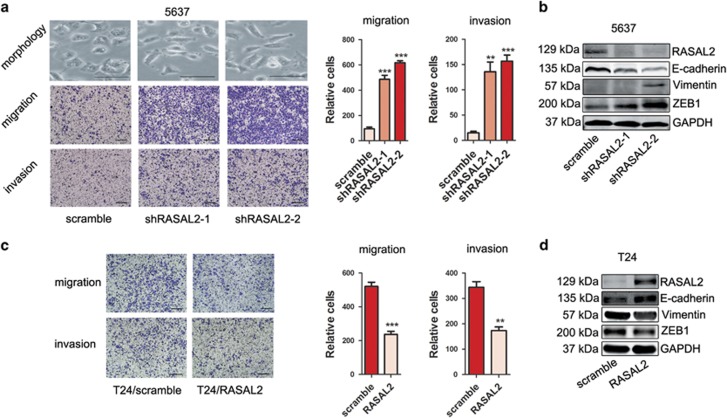
RASAL2 inhibits EMT of BCa cells. (**a**) Morphological change of 5637 cells transfected with RASAL2 shRNAs and scramble shRNA. The scale bar is 50*μ*m. Representative pictures and quantification analysis of migration and invasion assays in 5637 cells transfected with RASAL2 shRNAs and scramble shRNA. The scale bar is 100 *μ*m, **P<0.01, ***P<0.001 *versus* control. (**b**) Western blotting analysis of EMT markers (E-cadherin, vimentin and ZEB1) in 5637 sublines. (**c**) Representative pictures and quantification analysis of migration and invasion assays in T24 cells infected with RASAL2 lentivirus and scramble control. The scale bar is 100 *μ*m, **P<0.01, ***P<0.001 *versus* control. (**d**) Western blotting analysis of EMT markers (E-cadherin, vimentin and ZEB1) in T24 sublines. GAPDH was used as internal loading control

Current studies have reported that cancer stemness and EMT always appear coincidently, and EMT endows cells with a more CSC-like and mesenchymal phenotype.^19^ Indeed, 5637 cells revealed a cobblestone-like morphology; however, RASAL2-deficient 5637 cells displayed an elongated fibroblastoid appearance, as well as increased cell migration and invasion *in vitro* (Figures 3a, **P<0.01, ***P<0.001). Also, western blotting results showed that epithelial marker (i.e., E-cadherin) decreased but mesenchymal markers (i.e., vimentin, ZEB1) increased in RASAL2 knockdown 5637 cells compared with control (Figure 3b). In contrast, RASAL2 overexpression in T24 cells reversed EMT, and inhibited cell migration and invasion (Figures 3c and d, **P<0.01, **P<0.001). Similar results were shown in 253J and 253J-BV cells (Supplementary Figure 2). These data suggest that RASAL2 could suppress BCa migration and invasion through targeting EMT.

### RASAL2 regulates stemness and EMT via MAPK/SOX2 signaling pathway in BCa

**Figure 4:**
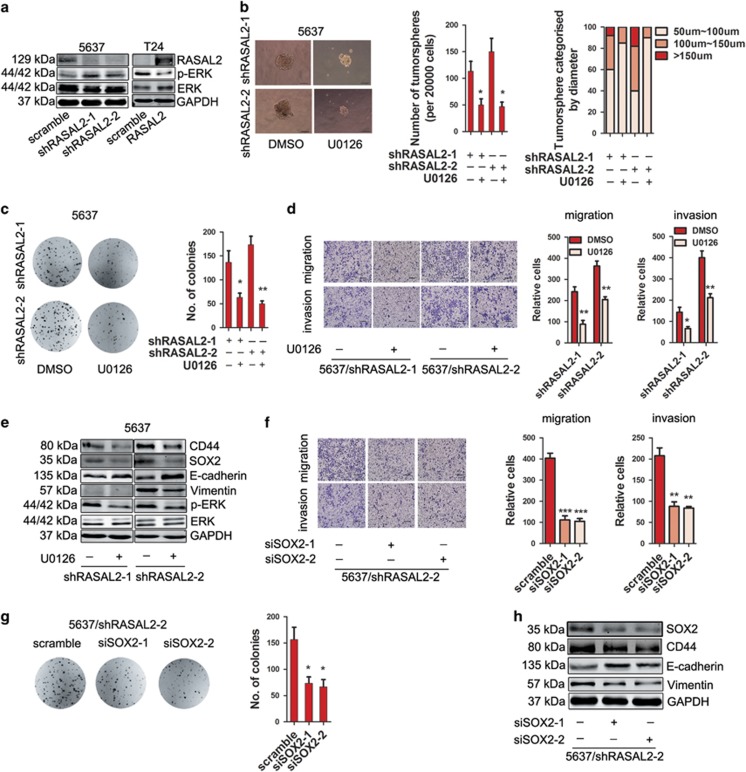
RASAL2 inhibits BCa stemness and EMT via ERK/SOX2 signaling pathway. (**a**) Western blotting analysis of p-ERK1/2 and total ERK1/2 in 5637 cells transfected with RASAL2 shRNAs and scramble shRNA, or T24 cells infected with RASAL2 lentivirus and scramble control. (**b**) Tumorsphere assay of 5637 cells transfected with RASAL2 shRNAs after treatment with MEK1/2 inhibitor U0126 (10 *μ*M). The tumorsphere number was counted and plotted, and percentage of tumorspheres with diameters <50 *μ*m, 50–100 *μ*m or >100 *μ*m was calculated and plotted. The scale bar represents 100 *μ*m, *P<0.05 *versus* DMSO. (**c**) Colony formation assay of 5637/shRASAL2 cells treated with MEK1/2 inhibitor U0126 (10 *μ*M) or DMSO. The colonies number was counted and plotted. *P<0.05, **P<0.01 *versus* DMSO. (**d**) Representative pictures and quantification analysis of migration and invasion abilities of 5637/shRASAL2 cells treated with MEK1/2 inhibitor U0126 (10 *μ*M) or DMSO. The scale bar is 100 *μ*m, *P<0.05, **P<0.01 *versus* DMSO. (**e**) Western blotting analysis of CD44, SOX2, E-cadherin, vimentin, P-ERK and ERK in 5637 cells transfected with RASAL2 shRNAs after treatment with MEK1/2 inhibitor U0126 (10 *μ*M). (**f**) Representative pictures and quantification analysis of migration and invasion assays in 5637/shRASAL2-2 cells transfected with SOX2 siRNAs and scramble siRNA. The scale bar is 100 *μ*m, **P<0.01, ***P<0.001 *versus* control. (**g**) Colony formation assay of 5637/shRASAL2-2 cells transfected with SOX2 siRNAs and scramble siRNA. The colonies number was counted and plotted. *P<0.05 *versus* control. (**h**) Western blotting analysis of CD44, SOX2, E-cadherin and vimentin in 5637/shRASAL2-2 cells transfected with SOX2 siRNAs and scramble siRNA. GAPDH was used as internal loading control

Next, it is the key to dissect the mechanisms of RASAL2 in regulating BCa stemness and EMT. As RASAL2 is a family member of RAS-GAP, we examined the activities of mitogen-activated protein kinase (MAPK) signaling. Indeed, we found that phosphorylated-ERK (p-ERK) levels increased in 5637 sublines with RASAL2 knockdown but decreased in T24 sublines with RASAL2 overexpression (Figure 4a). So we treated RASAL2-deficient 5637 cells with a specific MEK1/2 inhibitor U0126, and observed that these cells treated with U0126 exhibited a decrease of tumorsphere and colony formation (Figures 4b and c, *P<0.05, **P<0.01), a suppression of cell migration and invasion (Figure 4d and Supplementary Figure 3, *P<0.05, **P<0.01), as well as the reverse of EMT phenotype and suppression of stemness markers (i.e., CD44 and SOX2) expression (Figure 4e and Supplementary Figure 3). To further elucidate the specific roles of SOX2, we also applied siRNA strategy to knockdown SOX2 in RASAL2-deficient 5637 sublines, and found that siSOX2 could abolish the induction of stemness and EMT by RASAL2 loss (Figure 4h and Supplementary Figure 4), and significantly suppress colony formation, cell migration and invasion (Figures 4f and g and Supplementary Figure 4, *P<0.05, **P<0.01, ***P<0.001). These results may suggest that RASAL2 inhibits stemness and EMT in BCa via MAPK/SOX2 signaling pathway.

### RASAL2 modulates BCa cell tumorigenicity and distant metastasis *in vivo*

**Figure 5:**
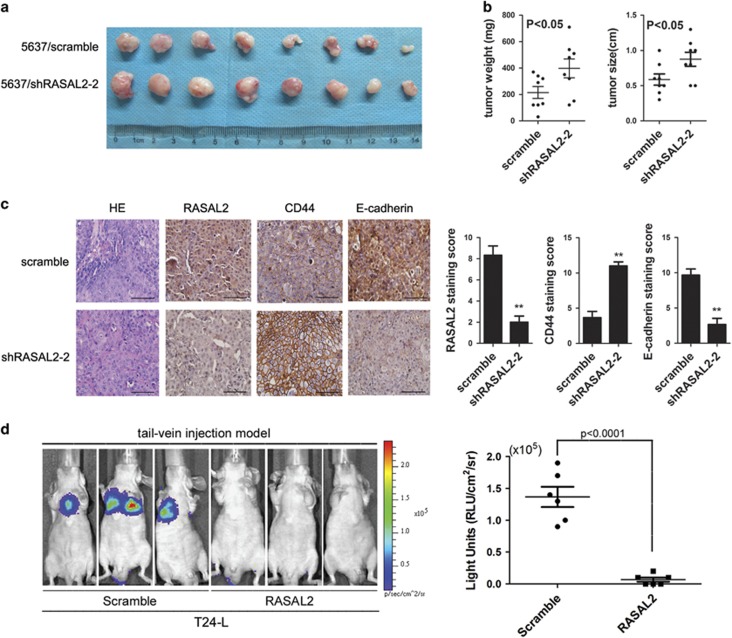
Enhanced tumorgenicity of RASAL2-deficient BCa cells along with the properties of stemness and mesenchymal characteristics. (**a** and **b**) Photograph pictures and quantification analysis of subcutaneous xenografts in nude mice implanted with 5637/shRASAL2-2 and 5637/scramble sublines (*n*=8). Xenografts weight (mg) and size (cm) were measured. (**c**) Immunohistochemistry staining of RASAL2, CD44 and E-cadherin in xenograft tissues from 5637/shRASAL2-2 and 5637/scramble xenograft tumors. The scale bar represents 100 *μ*m, **P<0.01 *versus* control. (**d**) Representative BLI images of athymic BALB/c nude mice implanted with T24L/scramble and T24L/RASAL2 cells by tail-vein injection, and quantification of light emission for lung metastatic lesion was calculated (*n*=6)

To further verify the tumor-suppressive role of RASAL2 in BCa *in vivo*, we established the subcutaneous xenograft using 5637 sublines. We observed that RASAL2 downregulation resulted in an increased tumor weight and size compared with control (Figures 5a and b). Furthermore, we also next compared the expression of E-cadherin and CD44 in the xenograft tissues by immunohistochemistry (IHC) staining. In consistent with our *in vitro* results, 5637/shRASAL2 tumors with lower RASAL2 expression presented a higher CD44 staining but a decreased E-cadherin staining compared with control (Figure 5c, **P<0.01).

Also, we further established the tail-vein injection metastasis model using T24-L sublines to explore the role of RASAL2 on BCa metastasis *in vivo.* As described in our previous studies,^20^ tail-vein injection of T24-L sublines could form lung metastasis, whereas overexpression of RASAL2 in T24-L sublines markedly abolished the incidence of lung metastasis *in vivo* (Figure 5d, *P*<0.0001). All these results suggested that RASAL2 could modulate tumorigenicity and distant metastasis along with modulation of stemness and EMT *in vivo*.

### RASAL2 is correlated with the status of stemness and EMT in BCa specimens

**Figure 6:**
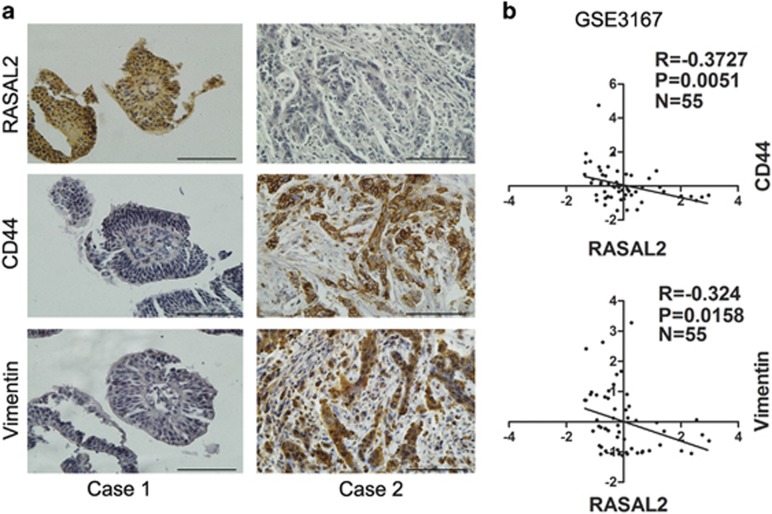
Correlation between RASAL2 and CD44 and EMT markers in human BCa tissues. (**a**) Representative pictures of RASAL2, CD44 and EMT markers (vimentin) immunohistochemistry in BCa tissues were shown. The scale bar represents 100 *μ*m. (**b**) Correlation between RASAL2 and CD44 or vimentin mRNA in BCa tissues from a public available microarray database (GSE3167, *n*=55)

We also utilized our clinical samples to strengthen our finding in cell lines and xenografts. Indeed, we found that higher CD44 and vimentin expression but lower E-cadherin expression was detected in MIBC than NMIBC (Supplementary Figure 1b, *P<0.05, **P<0.01). Also, there was a negative correlation between RASAL2 expression and the status of stemness and EMT in BCa tissues. As shown in Figure 6a, RASAL2 is negatively correlated with CD44 and vimentin expression (Pearson's correlation coefficient is −0.61 between RASAL2 and CD44, −0.53 between RASAL2 and vimentin), which was also supported by other microarray data (GSE3167) from NCBI GEO (Figure 6b). These data supported RASAL2 as a critical regulator in stemness and EMT of BCa.

## Discussion

Patients with MIBC or metastatic BCa have a very poor prognosis, and now fewer therapeutic strategies are available to prolong survival.^21^ Therefore, it is crucial and urgent to dissect the molecular mechanisms leading to BCa invasion and metastasis. Herein, we provide evidence that RASAL2 acts as a tumor suppressor in BCa, and modulates the phenotypes of cancer stemness and EMT through MAPK/SOX2 pathway.

RASAL2 belongs to the RAS GAP family members, in which neurofibromin (NF1) and DAB2IP have been most extensively studied. In most instances, *NF1* and *DAB2IP* behave as the classical tumor-suppressor genes.^12^ For example, our and other previous studies have demonstrated that DAB2IP has distinct cellular functions such as concurrently modulating different oncogenic pathways associated with cell proliferation, survival, apoptosis and metastasis.^22, 23, 24^ However, the expression and functions of RASAL2 in different cancer types remain largely unknown. Especially, the controversial effects of RASAL2 in breast cancer development have been reported,^16, 18^ indicating its role as a double-edged sword in certain condition.

Initially, RASAL2 was identified as a potential RAS GAP tumor suppressor in a functional cell-based screen.^25^ McLaughlin *et al.*^18^ further demonstrated that RASAL2 appeared to be more frequently silenced by epigenetic mechanisms in breast cancer, and RASAL2 suppression promoted breast tumor development as a consequence of activating K- and H-RAS based on the studies with human xenografts and genetically engineered mouse models. Moreover, in a luminal model of breast cancer, RASAL2 mutations promoted metastasis and correlated with recurrence and poor overall survival of patients with luminal B cancers. Instead, Feng *et al.*^16^ showed that RASAL2 was overexpressed in a subset of triple-negative or estrogen receptor-negative (ER-negative) breast tumors, and activated small GTPase RAC1 signaling to drive mesenchymal invasion and metastasis, indicating its context dependency in breast cancer. In this study, we found that RASAL2 was consistently downregulated either in NMIBC or MIBC tissues, and inversely correlated with pathological grades and clinical stages. Also, by performing gain-of-function and loss-of-function studies *in vitro* and *in vivo*, we demonstrated that RASAL2 could inhibit BCa stemness and EMT, which were critical for BCa invasion and metastasis. Moreover, to be consistent with the previous studies reported in luminal breast cancer and ovarian cancer,^17, 18^ we also observed that RASAL2 could modulate BCa cells stemness and EMT via MAPK pathway, in which the transcription factor SOX2 acted as an important bridge. Indeed, amount of studies have highlighted the central roles of SOX2 in maintaining stemness or cellular plasticity in cancer cells,^26, 27, 28, 29^ in particular, it has been reported that SOX2 could be regulated by MAPK signaling for maintaining side population or CSCs in human NMIBC.^30^

Taken together, our study revealed a novel mechanism of BCa invasion and metastasis, in which RASAL2 loss could facilitate BCa cell migration, invasion, stemness and tumorigenesis. Furthermore, our findings provide evidence for the critical roles of downstream MAPK/SOX2 signaling in BCa development. Therefore, RASAL2 could be a potential prognostic marker and drug target for BCa diagnosis and treatment.

## Materials and methods

### Cell culture and reagents

Human BCa 5637, T24, 253J, 253J-BV cells were purchased from the American Type Culture Collection (ATCC, Manassas, VA, USA), and cultured in RPMI-1640 medium for 5637 cells or DMEM medium for T24, 253J and 253J-BV cells supplemented with 10% fetal bovine serum at 37 °C aired with 5% CO_2_. Highly lung metastatic T24-L subline cells expressing luciferase were generated as described previously and cultured in DMEM medium supplemented with 10% fetal bovine serum and 600 mg/l G418 at 37 °C aired with 5% CO_2_.^20^ The MEK1/2 inhibitor U0126 and the antibiotic G418 were obtained from Sigma-Aldrich (St. Louis, MO, USA), and dissolved in DMSO and stored at −20 °C. The antibodies used were as follows: RASAL2 (rabbit, Abcam, Cambridge, UK), GAPDH (mouse, KangChen Bio-Tech, Shanghai, China), CD44 (mouse, Cell Signaling Technology, Beverly, MA, USA), SOX2 (rabbit, Cell Signaling Technology), E-cadherin (rabbit, Santa Cruz Biotechnology, Santa Cruz, CA, USA), Vimentin (rabbit, Cell Signaling Technology), ZEB1 (rabbit, Cell Signaling Biotechnology), p-ERK1/2 (Thr202/Tyr204) and ERK1/2 (rabbit, Cell Signaling Technology), p-MEK1/2 (Ser217/221) and MEK1/2 (rabbit, Cell Signaling Technology).

### Plasmid or siRNA transfection or lentiviral infection

RASAL2 shRNA or cDNA plasmids (GenePharma, Shanghai, China) were used to stably silence or overexpress the expression of RASAL2. RASAL2 shRNA backbone was pGPH1. The sequence of RASAL2 shRNAs was as follows: shRASAL2-1: 5′-CACCGCATGCATCTGTCATGCTTGATTCAAGAGATCAAGCATGACAGATGCATGCTTTTTTG-3′, shRASAL2-2: 5′-CACCGCCAAAGGCCTCTATAGATTCTTCAAGAGAGAATCTATAGAGGCCTTTGGCTTTTTTG-3′. siRNAs (RiboBio,Guangzhou, China) were used to transiently silence the expression of RASAL2 and SOX2. The sequence of siRNAs for RASAL2 and SOX2 was as follows: siRASAL2-1: 5′-TTTGCTCGTACAACCAGCA-3′, siRASAL2-2: 5′-GGATCGTTGTGGAGAGCAT-3′ siSOX2-1: 5′-CCAAGACGCTCATGAAGAA-3′, siSOX2-2: 5′-GGAGCACCCGGATTATAAA-3′. Both shRNA and siRNA were transfected with X-tremeGENE HP DNA or X-tremeGENE siRNA transfection reagents (Roche Diagnostics, Indianapolis, IN, USA) following the manufacturer's instructions. Lentivirus-overexpressing RASAL2 and scramble control were obtained from GeneCopoeia (Guangzhou, China), and viral supernatant was incubated with target cells for 12 h with 8 *μ*g/ml polybrene following the manufacturer's instructions.

### Colony formation assay

Cells were seeded into a six-well plate (1000 cells per well), and incubated with fresh medium in a humidified atmosphere at 37 °C with 5% CO_2_ for 14 days. The plates were washed by phosphate-buffered saline (PBS), fixed in 4% formalin, stained in crystal violet solution for 15 min and then washed with PBS to remove excess dye. The number of colony was counted for each sample.

### Tumorsphere formation assay

The cells were plated in six-well ultra-low attachment plates (20 000 cells per well) (Corning, Corning, NY, USA) in serum-free DMEM/F12 medium, then added with 20 ng/ml human EGF, 10 ng/ml human bFGF, and 2% B27 (Invitrogen, Carlsbad, CA, USA). Cells were incubated at 37 °C with 5% CO_2_. After 2 weeks, plates were analyzed for tumorsphere formation and counted by microscope.

### Migration and invasion assay

Migration and invasion were tested by Boyden chamber assay, obtained from Millipore (Schaffhausen, Switzerland). For migration, the cells were harvested and seeds into the upper chamber (8  *μ*m pore polycarbonate membrane filters) with 3000 cells per well in 0.3 ml serum-free RPMI-1640 for 5637 cells or DMEM for T24, 253J and 253J-BV cells, and 1 ml RPMI-1640 or DMEM medium containing 10% FBS was added to the lower chamber. After 12-h incubation, the upper surface of the chambers were wiped with a Q-tip and fixed with 4% formalin for 15 min, then stained with crystal violet for 15 min followed by washing three times with PBS. The cell number was counted in five random fields in the × 100 magnification. For the invasion assay, the upper chamber was coated with Matrigel (Sigma, St. Louis, MO, USA) and incubated in 37 °C with 5% CO_2_, 5 h later, 80 000 cells were seeded into the upper chamber in 0.3 ml serum-free RPMI-1640 for 5637 cells or DMEM for T24, 253J and 253J-BV cells. The plates were incubated for 36 h and the rest of the protocol conducted in a similar manner as the migration assay.

### Western blotting

Cells lysates were prepared with RIPA (50 mM Tris, PH 8.0), 150 mM NaCl, 0.1% SDS, 1% NP40 and 0.5% sodium deoxycholate) containing proteinase inhibitors (1% inhibitors cocktail and 1 mM PMSF) (Sigma). Proteins were separated by 12% SDS-PAGE, and transferred onto nitrocellulose membranes. After blocking with 5% non-fat dry milk in TBST, membrane was incubated with primary antibody at 4 °C overnight. Then washed by TBST, the membranes were incubated with fluorescent secondary antibodies (Licor, Rockford, IL, USA) at room temperature for 1 h. After washing by TBST, the membranes were scanned by Odyssey Detection system (Licor). Loading differences were normalized using GAPDH.

### Real-time RT-PCR

Total cellular RNA was extracted using RNAfast 200 reagents (Fastagen Biotechnology, Shanghai, China) and quantitated by absorbance at 260 nm. The RNA (2 *μ*g) sample was reversely transcribed with PrimeScript RT Master Mix, and quantitative PCR was performed with SYBR-Green PCR Master Mix (Takara Bio, Dalian, China) with the gene-specific primers: RASAL2, F: 5′-AGCAGAAAGGTCCCCTCGTAG-3′ R: 5′-AGGGTGAGGTATTTGCAGTGT-3′ GAPDH, F: 5′-ATGGGGAAGGTGAAGGTCGG-3′ R: 5′-GACGGTGCCATGGAATTTGC-3′. GAPDH was used as loading control.

### Tumor xenograft model

Male athymic nude mice were used according to the protocols approved by the ethical committee of Xi'an Jiaotong University. In all, 100 *μ*l serum-free RPMI-1640 medium containing Matrigel (1 : 1, v/v) with 1 × 10^6^ 5637 sublines (scramble or shRASAL2-2) were injected subcutaneously into both flanks. The mice were killed and xenografts were harvested at day 30, and tumors were weighed and measured for the tumor diameter, and then were stained by immunohistochemistry.

Tail-vein injection metastasis model were generated as described in previous studies.^20, 31^ Female athymic BALB/c nu/nu mice at age of 4–6 weeks were used according to the protocols approved by the ethical committee of Xi'an Jiaotong university. In all, 5 × 10^6^ T24-L cells were injected i.v. via the tail vein. Then, bioluminescence imaging (BLI) was performed to monitor lung metastases with injection of 450 mg/kg D-luciferin substrate (Biosynth, Naperville, IL, USA) in PBS into anesthetized mice.^20^

### Clinical specimens and immunohistochemistry

All the primary BCa tissues (*n*=132) were obtained from the Department of Urology, The First Affiliated Hospital of Xi'an Jiaotong University, Xi'an, China. All samples were used after written consent was obtained from patients. The IHC was performed using the EnVision system (Dako, Carpinteria, CA, USA), Tissue section were de-paraffinized, rehydrated and subjected to 5-min pressure cooker antigen retrieval methods, 10-min of endogenous enzyme block, incubated with the primary antibody overnight at 4 °C, 30-min Dako Cytomation EnVision-HRP reagent incubation for primary antibodies. Then, the signals were detected by diaminobenzidine (DAB) followed by hematoxylin counterstaining. The result was evaluated according to the intensity of the staining (0, 1+, 2+ and 3+) and the percentage of positive cells, which were separated by 0 (0%), 1 (1–25%), 2 (26–50%), 3 (51–75%) and 4 (76–100%). Finally, the result is considered by the staining score and the percentage of staining level negative (0 score), weak (1–4 score), moderate (5–8 score) and strong (9–12 score).

### Statistical analysis

All the statistical analyses were performed by GraphPad Prism version 5.0 software (GraphPad Software, La Jolla, CA, USA). All data were reported as mean±S.E.M., and the differences between two groups were compared by the two-tailed Student's *t*-test. For analyzing gene expression profiling and correlation, we used the data set GSE3167^32^ and GSE31684,^33, 34^ which were downloaded from NCBI GEO database. *P<0.05 was considered statistically significant.

## Figures and Tables

**Table 1:** Distribution by tumor characteristics for our patients with bladder cancer

Mean age at diagnosis (years)	68.6
*Gender (*n*)*	
Male	108
Female	24
	
*Stage (*n*)*	
Ta–1	66
T2–4	66
	
*Grade (*n*)*	
PUNLMP	11
Low grade	40
High grade	81
Total (*n*)	132
