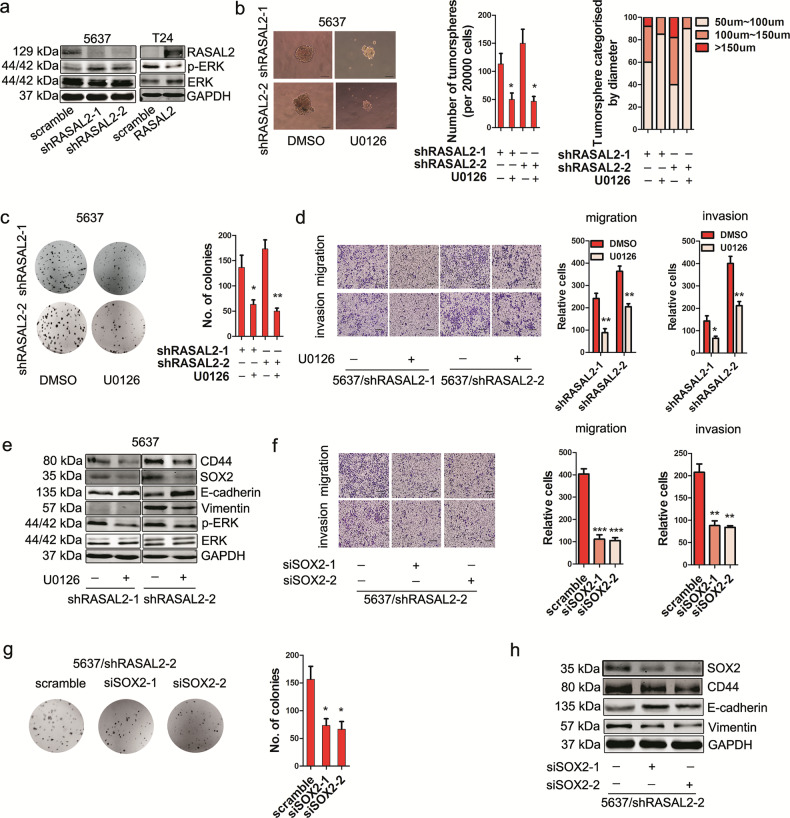# Correction: RASAL2, a RAS GTPase-activating protein, inhibits stemness and epithelial-mesenchymal transition via MAPK/SOX2 pathway in bladder cancer

**DOI:** 10.1038/s41419-026-08630-3

**Published:** 2026-04-17

**Authors:** Ke Hui, Yang Gao, Jun Huang, Shan Xu, Bin Wang, Jin Zeng, Jinhai Fan, Xinyang Wang, Yangyang Yue, Shiqi Wu, Jer-Tsong Hsieh, Dalin He, Kaijie Wu

**Affiliations:** 1 Department of Urology, The First Affiliated Hospital of Xi’an Jiaotong University,, Xi’an, China; 2 Department of Urology, Shaanxi Provincial People’s Hospital, Xi’an, China; 3 Department of Urology, University of Texas Southwestern Medical Center, Dallas, TX USA

Correction to: *Cell Death & Disease* 10.1038/cddis.2017.09, published online 09 February 2017

Recently, we found that the representative pictures of shRASAL2-2 cells in the invasion assay from panel A of Fig. 3, NC cells in the invasion assay from panel F of Fig. 4, and representative pictures from panel C and panel G of Fig. 4 (the red rectangle) were misused. So, now we renewed them with the new pictures.

**original Fig. 3** 
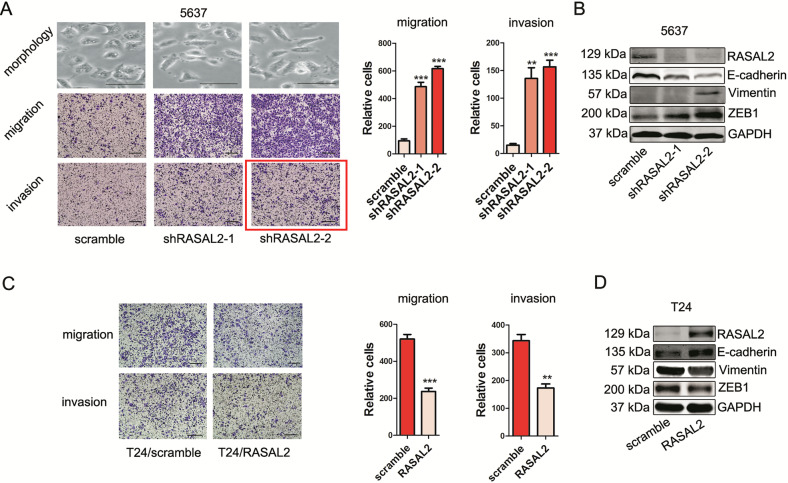


**corrected Fig. 3** 
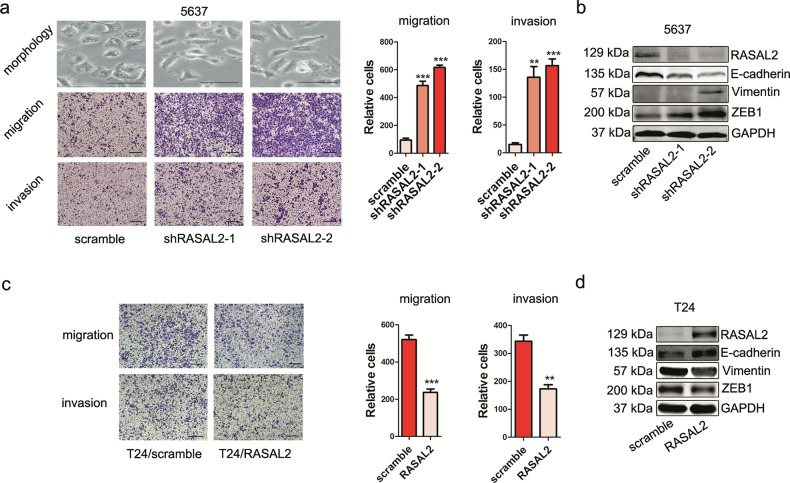


**original Fig. 4** 
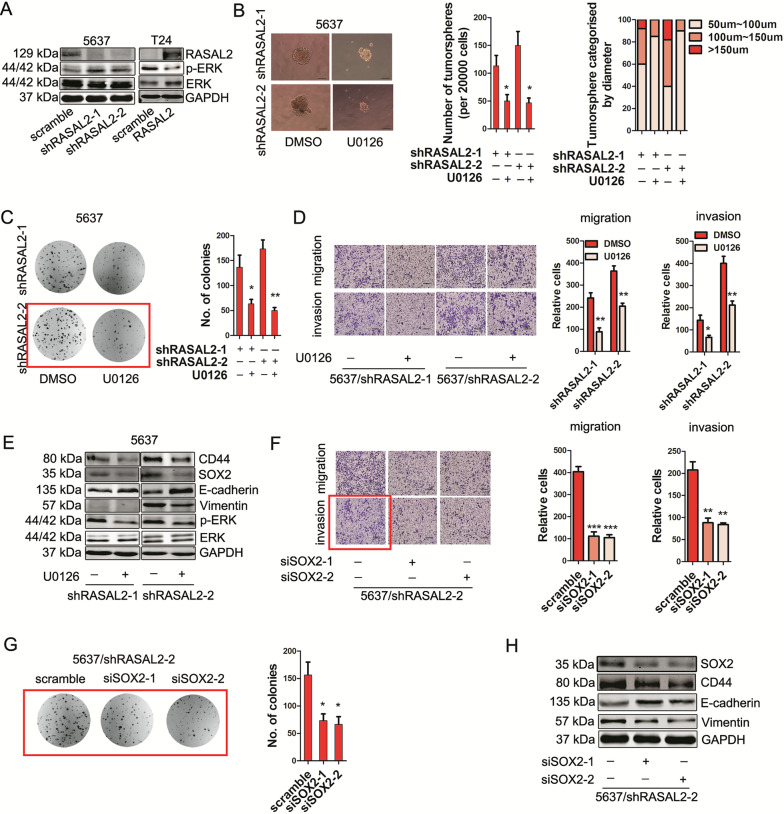


**corrected Fig. 4**