# Supplementary figures and images for: RASAL2, a RAS GTPase-activating protein, inhibits stemness and epithelial–mesenchymal transition via MAPK/SOX2 pathway in bladder cancer
Source: Cell Death Dis. 2017 Feb 9;8(2):e2600–. doi: 10.1038/cddis.2017.9 (PMC5386500; doi:10.1038/cddis.2017.9)

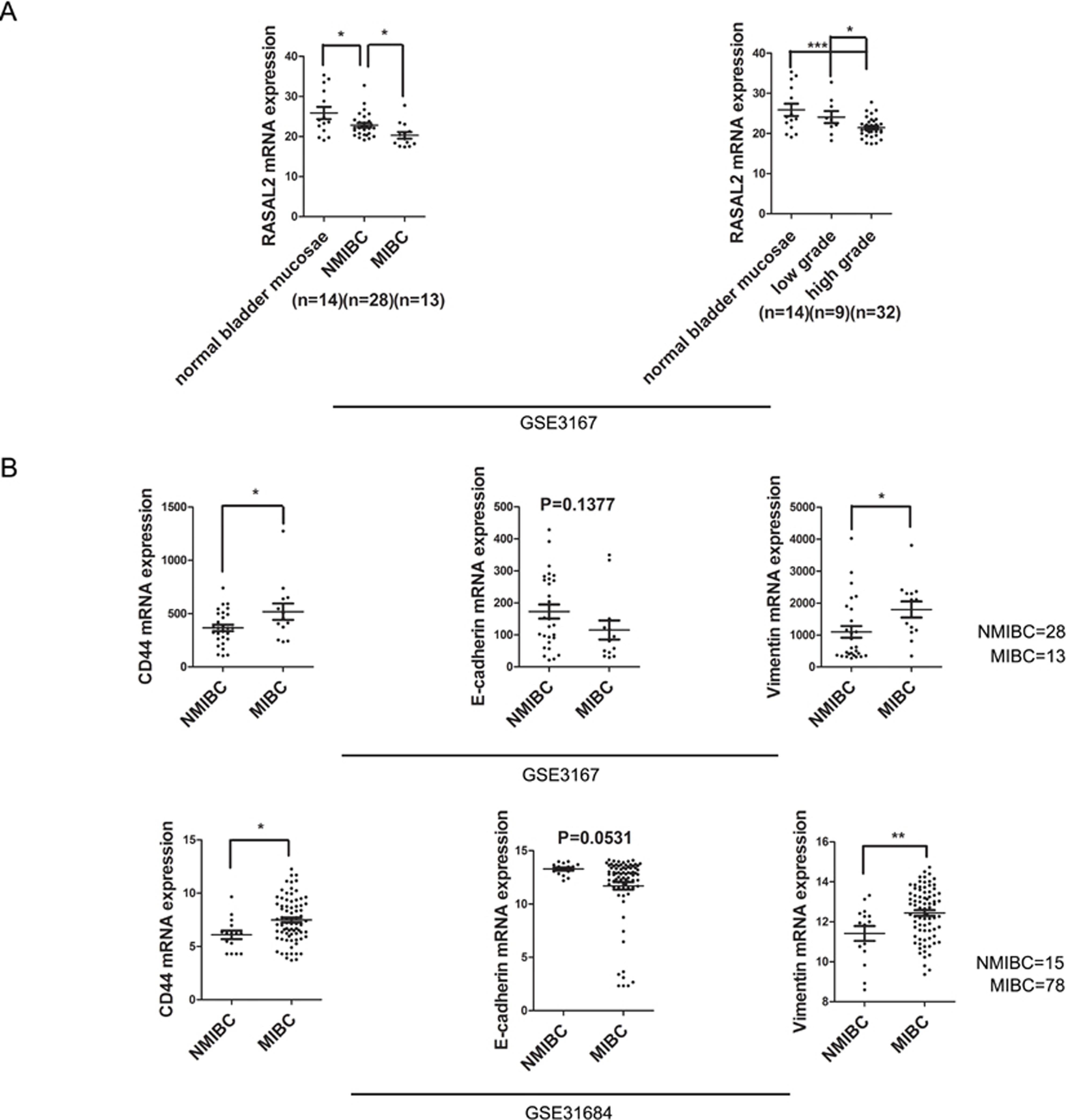

Supplement: Supplementary Figure S1 [file cddis20179x1.tif]

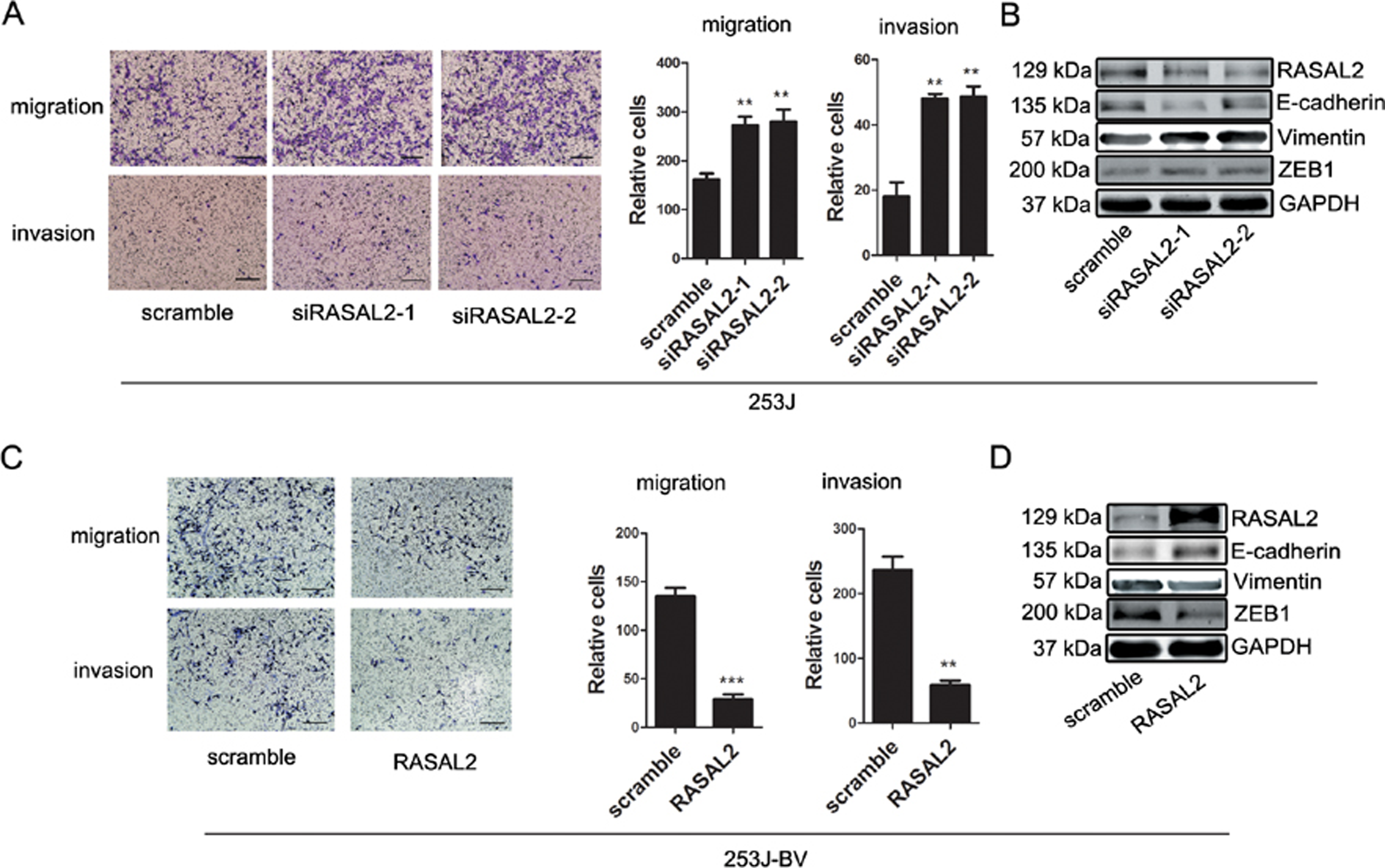

Supplement: Supplementary Figure S2 [file cddis20179x2.tif]

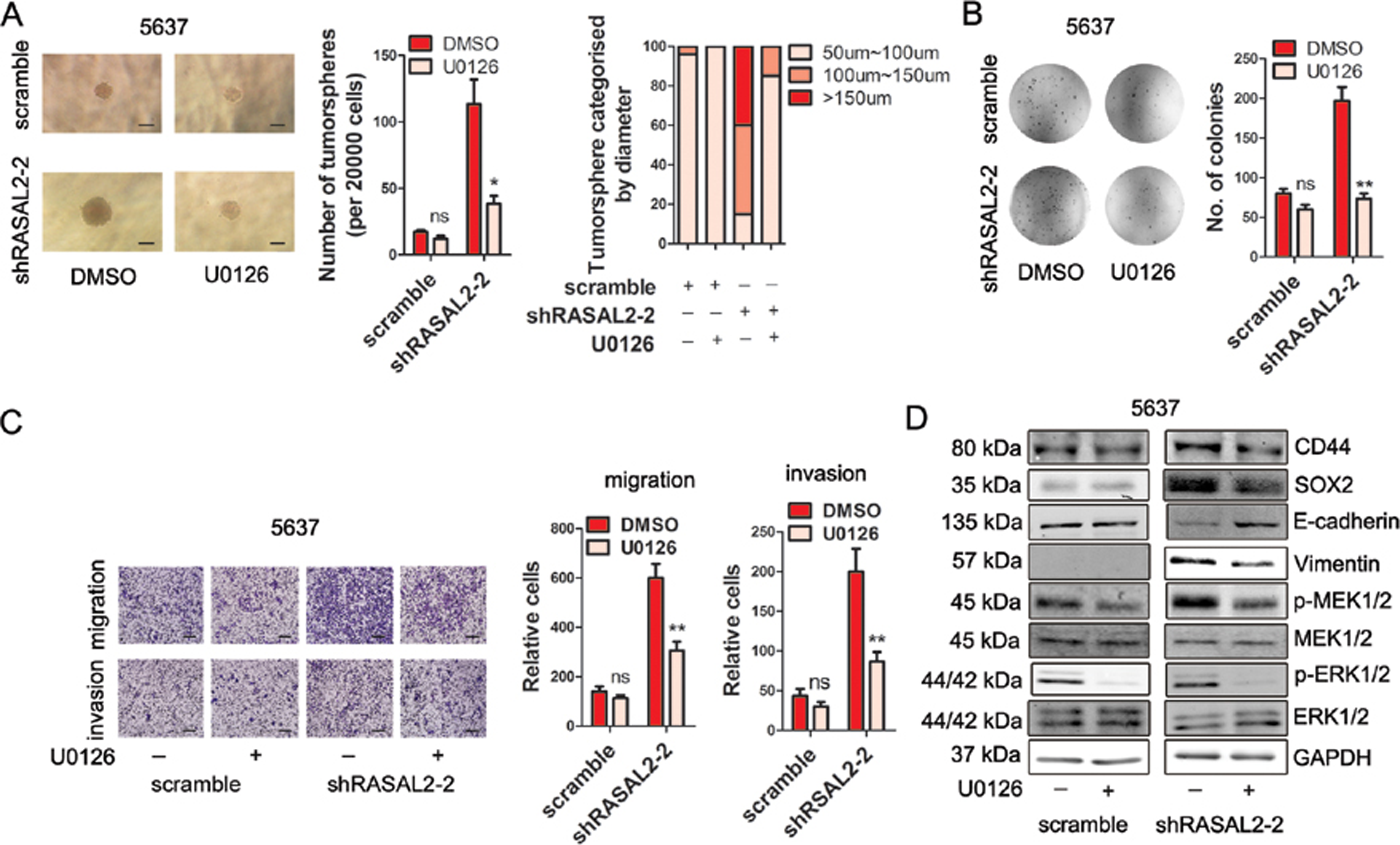

Supplement: Supplementary Figure S3 [file cddis20179x3.tif]

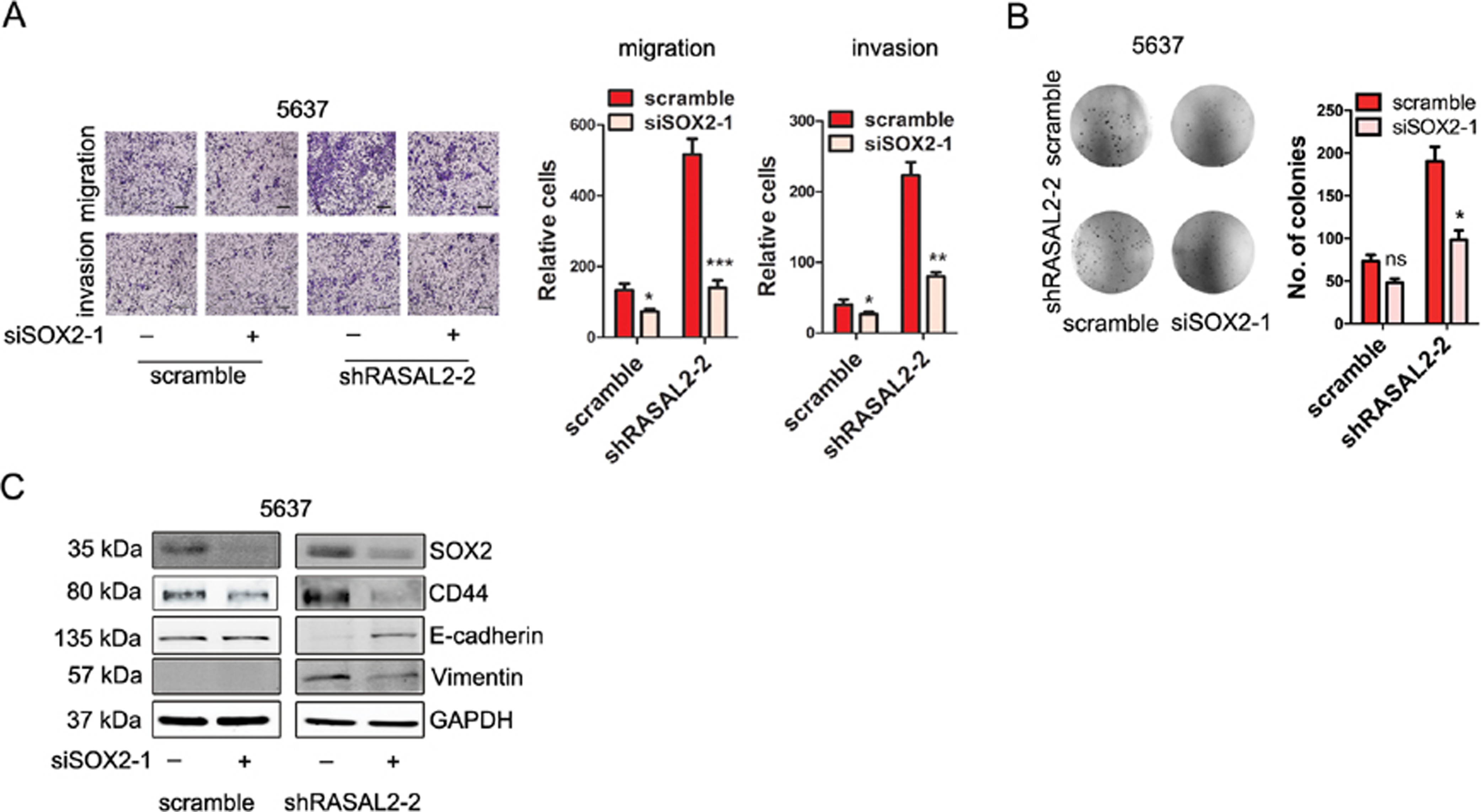

Supplement: Supplementary Figure S4 [file cddis20179x4.tif]
