# Supplementary material for: RASAL2, a RAS GTPase-activating protein, inhibits stemness and epithelial–mesenchymal transition via MAPK/SOX2 pathway in bladder cancer
Source: Cell Death Dis. 2017 Feb 9;8(2):e2600–. doi: 10.1038/cddis.2017.9 (PMC5386500; doi:10.1038/cddis.2017.9)
Supplement: Supplementary Material [file cddis20179x5.doc]

**Figure legends:**

**Supplementary Fig.1 Expression levels of RASAL2, CD44 and EMT markers in human BCa tissues.** A, Analysis of RASAL2 mRNA in normal bladder urothelium and BCa tissues from a public available microarray database (GSE3167), **P* < 0.05 and ****P* < 0.001. B, Analysis of CD44, E-cadherin and vimentin mRNA levels in NMIBC and MIBC tissues from two different public available microarray database (GSE3167 and GSE31684).

**Supplementary Fig.2 RASAL2 modulated EMT in different BCa cells.** A, Representative pictures and quantification analysis of migration and invasion assays in 253J cells transfected with RASAL2 siRNAs and scramble siRNA. The scale bar is 100 μm, **P* < 0.05 versus control. B, Western blotting analysis of EMT markers (E-cadherin, vimentin and ZEB1) in 253J cells transfected with RASAL2 siRNAs and scramble siRNA. C, Representative pictures and quantification analysis of migration and invasion assays in 253J-BV cells infected with RASAL2 lentivirus and sramble control. The scale bar is 100 μm, ***P* < 0.01 and ****P* < 0.001 versus control. D, Western blotting analysis of EMT markers (E-cadherin, vimentin and ZEB1) in 253J-BV cells infected with RASAL2 lentivirus and sramble control. GAPDH was used as internal loading control.

**Supplementary Fig.3 RASAL2 inhibits BCa stemness and EMT via MAPK/ERK pathway.** A, Tumorsphere assay of 5637 cells transfected with scramble and RASAL2-2 shRNA after treatment with MEK1/2 inhibitor U0126 (10 μM). The tumorsphere number was counted and plotted, and percentage of tumorspheres with diameters < 50 μm, 50-100 μm, or > 100 μm was calculated and plotted. The scale bar represents 100 μm, **P*<0.05. B, Colony formation assay of 5637/scramble and 5637/shRASAL2-2 cells treated with MEK1/2 inhibitor U0126 (10 μM) or DMSO. The colonies number was counted and plotted. ***P*<0.01. C, Representative pictures and quantification analysis of migration and invasion abilities of 5637/scramble and 5637/shRASAL2-2 cells treated with MEK1/2 inhibitors U0126 (10 μM) or DMSO. The scale bar is 100μm, ***P*<0.01. D, Western blotting analysis of CD44, SOX2, E-cadherin, vimentin, p-MEK, MEK, p-ERK and ERK in 5637/scramble and 5637/shRASAL2-2 cells treated with MEK1/2 inhibitor U0126 (10 μM) or DMSO.

**Supplementary Fig.4 RASAL2 inhibits BCa stemness and EMT by downregulating SOX2.** A, Representative pictures and quantification analysis of migration and invasion abilities of 5637/scramble and 5637/shRASAL2-2 cells transfected with SOX2 siRNA-1 and scramble siRNA. The scale bar is 100μm. **P*<0.05, ***P*<0.01,****P*<0.001. B, Colony formation assay of 5637/scramble and 5637/shRASAL2-2 cells transfected with SOX2 siRNA-1 and scramble siRNA. The colonies number was counted and plotted, **P*<0.05. C, Western blotting analysis CD44, SOX2, E-cadherin and vimentin in 5637/scramble and 5637/shRASAL2-2 cells transfected with SOX2 siRNA-1 and scramble siRNA. GAPDH was used as internal loading control.
